# New Medical Journal

**Published:** 1848-10

**Authors:** 


*New Medical Journal.*—A medical journal has just been commenced at Columbus, Ohio, called the *Ohio Medical and Surgical Journal,* edited by John Butterfield, M. D., Professor of the Practice of Medicine in the Starling Medical College. It is a bi-monthly of ninety-six pages. Subscription price two dollars. We tender our friend, the editor, our warmest sympathies in his prospective afflictions. *Haud inexpertus loquor.*

